# Atlas of Venereal and Skin Diseases

**Published:** 1890-02

**Authors:** 


ATLAS OF VENEREAL AND SKIN DISEASES, comprising original illustrations and selections from the plates of Prof. M. Kaposi, of Vienna; Mr. J. Hutchinson, of London; Prof. I. Neumann, of Vienna; Profs. A. Fournier and Hardy; and Drs. Ricord, Cullerier and Vidal, of Paris; Prof. Leloir, of Lille; Dr. Unna, of Hamburg; Dr. Silva Araujo, of Rio Janeiro; Dr. P. A. Morrow, of New York; Dr. A. R. Robinson, of New York; Dr. E. L. Keyes, of New York; Dr. J. Nevins Hyde, of Chicago; Dr. Henry G. Pifford, of New York, and others. With original text by Prince A. Morrow, A. M., M. D., Clinical Professor of Venereal Diseases, formerly Clinical Lecturer on Dermatology, in the University of the City of New York; Surgeon to Charity Hospital, New York. Wm. Wood & Co., 1889.

This work is composed of fifteen *fasciculi*, which were issued successively. Type is large and clear. There are numerous plates which, with a few exceptions, present the sacrifice of accurate reproduction to too vivid coloring. The text is concise, clear, and the author has embodied practically all the late advances in this specialty. The style is good, and condensation has proven an advantage. Were the work published without the extensive plates it would be one of the books which every physician should have.

The duality theory of venereal sores, and its evolution, is first discussed. Mentions as many as five varieties of chancroid. Says auto-inoculability is no proof of the disease, as the virus of chancroid can be generated *de novo*.

With others, does not admit Lustgarten's, so-called, discovery of the specific bacillus of syphilis. "Superficially eroded chancre most common." "Diphtheroid chancre" first described by the author—"rare." Consists of glistening, grayish-white, leathery coating, simulating diphtheritic exudation. "Moist and glistening, no appreciable secretion, not eroded, no induration." Detachment leaves bleeding base. Syphilitic bubo may suppurate, but always suggests "mixed" sore.

Results show excision of initial sore and of implicated glands to be worthless, but is indicated as removing source of infection for others. Best treatment of chancroid is the "expectant." Instead of the usual four varieties of papular he makes two divisions, viz.: "Miliary and lenticular," with papulo-squamous and moist papular as subdivisions. Gives two varieties of erythematous syphilide—the macular and the papular, the latter an exaggerated development of the former. Retains the term syphilitic roseola (for erythematous), which has been abandoned by many. Describes some forms of papular not mentioned in most of the dermatological works. Does not mention an especial tendency of the "papulo-squamous" to affect palms and soles. Does not classify the (rare) vesicular syphilide, putting it in the general class of pustular syphilides. Modern division into stages is made wholly on pathological grounds, not with reference to time.

Follows the continental view as regards the influence of the father, of the mother, and of stage of the disease in producing hereditary syphilis. In an appendix gives voluminous *résumé* of treatment for syphilis, without an opinion as to relative value of the different plans. Says plasters containing resorcin, hydronaphthol, subiodide of bismuth, or other like remedies, have been found equally efficient with mercurials as local applications. Does not mention the salicylate of mercury, which many German authors praise as an intra-muscular injection.

In dermatology the author follows, in the main, the classification of the American Dermatological Association. Includes the exanthemata, and describes typhus and typhoid fevers, giving plates of eruptions. The plate representing measles is very good.

Says many other authors have pointed out the connection between the erythemata and hepatic and renal disorders.

Plate xlv, representing eczema, is very good. Claims to have called attention several years ago to spinal sinapisms, as valuable in treatment of genital and anal eczema. Crocker (of London) mentions this, for any obstinate form of the disease, and seems to consider himself the originator. Mentions, as quite groundless and controverted by every day experience, the common belief that infantile, eczematous disease should be left alone, lest internal complications follow a cure! Recognizes Unna's "eczema seborrhoicum," as a distinct variety. Devotes only a few lines to simple impetigo, saying, most authors now regard these conditions as pustular eczema.

"Drug eruptions," upon which the author has written a book, are described. Describes that disease composed of such a variety of elements—dermatitis, herpitiformis, of Duhring—now generally admitted by dermatologists.

Mentions "purpura thrombotica," (of Hutchinson) due to capillary thrombosis. (Hutchinson is noted for his large nomenclature, and variety of subdivisions.) Dr. Morrow does not express an opinion as to the identity or non-identity of lichen planus and lichen ruber, and describes them separately, following the majority of American dermatologists.

Plates representing lichen planus are of no use. Figure 2 of plate lviii shows very well Neumann's case of lichen moniliformis—as regards the pearl-like bands.

Regards lanugo hairs, so commonly found in sebaceous plugs (comedones), as responsible for the condition through mechanically obstructing exit of sebaceous matter.

Mentions probability of two varieties of alopecia areata; one contagious and presumably due to a parasite, the other due to "nervous shock." This is the view of some recent foreign writers. Thinks there are so many and striking analogies between lupus erythematosus and lupus vulgaris that they cannot properly be considered as distinct and independent diseases. (Since the discovery of the tubercular nature of lupus vulgaris the diseases have come to be considered, by many, absolutely dissimilar and "lupus" erythematosus a misnomer.)

Under the title "Tuberculosis Papillomatosa Cutis" is described a case at Charity Hospital which puzzled all who saw it, until the diagnosis was finally made by the microscope.

Plates representing leprosy seem good. Says the lepra bacillus is now generally recognized. Thinks parents probably transmit only hereditary tendency to leprosy. M. B. H.

